# The effects of inflammation on connexin 43 in chronic Chagas disease cardiomyopathy

**DOI:** 10.3389/fimmu.2024.1440662

**Published:** 2024-07-29

**Authors:** Breno Cardim Barreto, Maria Vitória Gomes das Neves, Carine Machado Azevedo Cardoso, Cássio Santana Meira, Pâmela Santana Daltro, Cláudio Pereira Figueira, Girlaine Café Santos, Daniela Nascimento Silva, Fábio Távora, João David de Souza Neto, Simone Garcia Macambira, Paul D. Lampe, Keyla Cristiny da Silva Coutinho, Tais Hanae Kasai Brunswick, Ricardo Ribeiro dos Santos, Antônio Carlos Campos de Carvalho, Milena Botelho Pereira Soares

**Affiliations:** ^1^ Gonçalo Moniz Institute, Oswaldo Cruz Foundation (IGM-FIOCRUZ/BA), Salvador, Brazil; ^2^ Department of Biochemistry and Biophysics, Federal University of Bahia (UFBA), Salvador, Bahia, Brazil; ^3^ SENAI Institute of Innovation in Health Advanced Systems (CIMATEC ISI SAS), University Center SENAI/CIMATEC, Salvador, Bahia, Brazil; ^4^ Messejana Heart and Lung Hospital, Fortaleza, Brazil; ^5^ Translational Research Program, Fred Hutchinson Cancer Center, Seattle, WA, United States; ^6^ Biophysics Institute Carlos Chagas Filho, Federal University of Rio de Janeiro, Rio de Janeiro, Brazil

**Keywords:** Chagas disease, cardiomyopathy, connexin 43, arrhythmias, inflammation

## Abstract

**Background:**

Cardiac arrhythmias are the main cause of sudden death due to Chronic Chagasic Cardiomyopathy (CCC). Here we investigated alterations in connexin 43 (Cx43) expression and phosphorylation in cardiomyocytes as well as associations with cardiac arrhythmias in CCC.

**Methods:**

C57Bl/6 mice infected with *Trypanosoma cruzi* underwent cardiac evaluations at 6 and 12 months after infection via treadmill testing and EKG. Histopathology, cytokine gene expression, and distribution of total Cx43 and its phosphorylated forms Cx43^S368^ and Cx43^S325/328/330^ were investigated. Human heart samples obtained from subjects with CCC were submitted to immunofluorescence analysis. *In vitro* simulation of a pro-inflammatory microenvironment (IL-1β, TNF, and IFN-γ) was performed in H9c2 cells and iPSC-derived cardiomyocytes to evaluate Cx43 distribution, action potential duration, and Lucifer Yellow dye transfer.

**Results:**

Mice chronically infected with *T. cruzi* exhibited impaired cardiac function associated with increased inflammation, fibrosis and upregulated IL-1β, TNF, and IFN-γ gene expression. Confocal microscopy revealed altered total Cx43, Cx43^S368^ and Cx43^S325/328/330^ localization and phosphorylation patterns in CCC, with dispersed staining outside the intercalated disc areas, i.e., in lateral membranes and the cytoplasm. Reduced co-localization of total Cx43 and N-cadherin was observed in the intercalated discs of CCC mouse hearts compared to controls. Similar results were obtained in human CCC heart samples, which showed Cx43 distribution outside the intercalated discs. Stimulation of human iPSC-derived cardiomyocytes or H9c2 cells with IL-1β, TNF, and IFN-γ induced alterations in Cx43 localization, reduced action potential duration and dye transfer between adjacent cells.

**Conclusion:**

Heart inflammation in CCC affects the distribution and phosphorylation pattern of Cx43, which may contribute to the generation of conduction disturbances in Chagas disease.

## Introduction

Considered by the World Health Organization (WHO) to be both a neglected disease and a major public health problem, Chagas disease, caused by the protozoan *Trypanosoma cruzi*, is endemic to Latin America and present mainly in low-income regions (1). Due to the migration of infected individuals to other regions of the world for socioeconomic reasons (2), the disease has also spread to other continents. Currently, it is estimated that 25,000 people acquire the disease annually, and approximately 10,000 people die worldwide each year due to Chagas disease (1).

The course of disease begins with an acute phase, which corresponds to the onset of infection and parasite spread throughout the body, while the chronic phase mainly affects the digestive system and/or the heart (3). Chronic Chagasic Cardiomyopathy (CCC), the most common clinical manifestation of Chagas disease, displays high morbidity and mortality (4), with cardiac arrhythmias being the main cause of sudden death in affected patients (5, 6).

Cardiac arrhythmias have been associated with alterations in the function and distribution of connexin 43 (Cx43) in cardiac cells (7). Cx43 is the main protein responsible for forming gap junction channels located in the intercalated discs between adjacent cardiac cells (8). Gap junctions play an important role in synchronizing rhythmic contractions and the maintenance of cardiac homeostasis through the exchange of small metabolites (9). Previous studies demonstrated that cardiomyocytes infected by *T. cruzi* present impaired cell-cell communication due to reduced Cx43 expression (9–12). Alterations in Cx43 have also been observed in arrhythmogenic cardiac diseases of other etiologies, such as ischemic and bacterial myocarditis (13–15).

The precise regulation of gap junctions is crucial for cell-cell communication and the proper propagation of electrical signals between cardiomyocytes. The phosphorylation of Cx43 is critical for regulating Cx43 activity, as well as this protein’s lifespan, structure, and localization (7). Among the phosphorylation sites, serines 368 (S368) and the triplet 325/328/330 (S325/328/330) have been well investigated, demonstrating their role in the regulation of Cx43 function (13, 15, 16). While Cx43^S368^ is involved in gap junction inactivation and disassembly, which contributes to the electrical uncoupling of cardiomyocytes, Cx43^S325/328/330^ has been associated with gap junction assembly (13, 16–18).

The importance of Cx43 to proper electrical activity in the heart, coupled with the fact that no specific treatments exist for CCC, highlights the need to further our understanding of the role played by Cx43 alterations and investigate relevant relationships with arrhythmias in Chagas disease. Therefore, the present study aimed to evaluate the expression and distribution of total Cx43, as well as its phosphorylated forms, in mice and the hearts of CCC patients. Additionally, we performed an *in vitro* investigation into the role of the pro-inflammatory chagasic microenvironment in Cx43 expression.

## Materials and methods

### Animals

Four-week-old male C57BL/6 mice were used for *T. cruzi* infection and as uninfected controls. All animals were obtained from the animal care facilities of the Biotechnology and Cell Therapy Center, São Rafael Hospital (CBTC-HSR), and the Gonçalo Moniz Institute, Oswaldo Cruz Foundation (IGM-FIOCRUZ), and kept at room temperature (20 ± 2°C) under controlled humidity (50%) conditions. Food and water were provided *ad libitum* and all animals were exposed to constant 12-hour light-dark cycles. All procedures were approved by the Animal Use Ethics Committee of the São Rafael Hospital (Protocol 011/18) and the Gonçalo Moniz Institute (Protocol 17/2017).

### *Trypanosoma cruzi* infection and experimental design

Mice were infected by intraperitoneal (i.p) injection of saline solution (100 µL) containing 10^3^ trypomastigotes of *T. cruzi* (Colombian strain), obtained from cultures of LCC-MK2 cells previously infected with *T. cruzi*. Parasitemia was assessed at different time points after infection using a standard protocol (19). All animals were evaluated for cardiac function (exercise testing and electrocardiogram) at 6 or 12 months after *T. cruzi* infection to compare groups of uninfected (n=10) and infected animals (n = 15) at each infection time. Following these evaluations, animals were euthanized using xylazine (50 mg/kg) and ketamine (100 mg/kg) via intraperitoneal, and murine hearts were collected to perform histopathological and immunofluorescence analysis, evaluate gene expression, and undergo transmission electron microscopy (**Supplementary Figure S1**).

### Treadmill testing

Exercise performance was evaluated by placing each mouse on a treadmill in a chamber (LE 8700; Panlab, Barcelona, Spain). Treadmill speed and shock intensity (mA) were controlled by potentiometer (LE 8700-treadmill control, Panlab). The initial speed was 6 cm/s, with increases of 6 cm/s every five minutes until reaching exhaustion. Exhaustion was considered following an animal’s permanence for a period of 10 seconds on the electrified stainless-steel grid that served as a stimulus to perform the activity. The parameters evaluated were walking distance and total exercise time (20).

### Electrocardiographic analysis

Mice were submitted to inhalational anesthesia with isoflurane (0.5 to 2%) to acquire electrocardiographic data. Electrocardiographic (EKG) records were obtained using the BioAmp Powerlab system (PowerLab 2\20, ADInstruments, Castle Hill, NSW, Australia) via bipolar leads I and II. The obtained data was then analyzed using LabChart7 software (PowerLab), applying filters in the range of 1 to 100 Hz to minimize environmental signal disturbances, with a sampling rate of 1 kHz. The parameters evaluated on EKG included heart rate, PR interval, P wave duration, QT interval, corrected QT interval (QTc), and the presence of arrhythmia. Wave durations (ms) and heart rate were automatically calculated by the software. QTc was calculated as the ratio of the QT interval to the square root of the RR interval (Bazett’s formula) (20, 21).

### Histopathological and morphometric analyses

Following euthanasia, murine heart samples were fixed in 4% formalin for paraffinization and the preparation of histological sections. The quantification of inflammatory cells and the percentage of fibrotic tissue were performed on slides under bright field microscopy following staining with hematoxylin-eosin (H&E) and Sirius red, respectively. Images were captured using a CoolSnap digital camera adapted to an AX-70 microscope (Olympus) and analyzed using Image-Pro Plus software, version 5.0 (Media Cybernetics). To quantify the number of inflammatory infiltrate cells, 5 fields/animal were captured from HE-stained slides at 400x magnification. The extent of fibrosis was estimated in Sirius red-stained heart sections using the same program by comparing areas of fibrotic and non-fibrotic tissue in 10 fields per animal at 200x magnification (21). The micrography analyses were done blinded.

### Gene expression analysis by RT-qPCR

RNA was extracted from murine heart tissue using TRIzol reagent (Invitrogen, Carlsbad, CA), with concentrations determined by photometric measurement. A High-Capacity cDNA Reverse Transcription Kit (Applied Biosystems, Foster City, CA) was used to synthesize cDNA from 1 μg of RNA in accordance with the manufacturer’s recommendations. cDNA synthesis and RNA expression analysis were performed by Real-Time PCR using TaqMan Gene Expression Assays for *Gja1* (Mm00439105_m1), *Tnf* (Mm00443258_m1), *Il1b* (Mm0043228_m1) and *Ifng* (Mm01168134_m1). All reactions were run in duplicate on an ABI 7500 Real Time PCR System (Applied Biosystems) under standard thermal cycling conditions. A non-template control (NTC) and non-reverse transcription controls (No-RT) were also included. Samples were normalized with *Hprt* (endogenous control). The threshold cycle (2-ΔΔCt) method of comparative PCR was used to analyze the obtained results (22).

### Human heart samples

Tissue samples of explanted hearts were obtained from the heart transplant service of Hospital de Messejana in Fortaleza (Ceará-Brazil). Left ventricular sections were obtained from the explanted hearts of patients with CCC (n = 3). As a control, a sample was obtained from the heart of a patient without cardiomyopathy who died from stomach cancer (n = 1) (**Supplementary Table S1**). Sections were processed and then subsequently subjected to immunofluorescence analysis. The local institutional review board of the Hospital de Messejana approved the present study protocol (Approval number: 3.255.044).

### Cardiac tissue immunofluorescence

Sections of paraffin-embedded hearts fixed in formalin were used to detect the expression and distribution of total Cx43, as well as phosphorylated Cx43^S368^ and Cx43^S325/328/330^ by immunofluorescence. Heart sections were incubated overnight with anti-total Cx43 (1:50; Santa Cruz Biotechnology, Santa Cruz, CA; SC-9059), anti-Cx43^S368^ (1:100; Thermo Fisher Scientific; 48-3000), anti-Cx43^S325/328/330^ (1:2000) (23) and N-cadherin (1:100; Thermo Fisher Scientific; 33-3900) antibodies, at 4°C. Next, secondary anti-rabbit IgG AlexaFluor 488 conjugated antibody (1:1000; Life Technologies; A21441) and Wheat Germ Agglutinin (1:1000; WGA - AlexaFluor 594; W11262), or anti-mouse IgG AlexaFluor 568 conjugated (1:1000; Life Technologies, A10037) were added, followed by a 1-hour incubation period at room temperature. Slides were mounted using Vectashield mounting medium with DAPI (Vector Laboratories). Images were obtained using a TCS SP8 spectral confocal microscope (Leica) and analyzed using ImagePro version 7.0 software (Media Cybernetics). The micrography analyses were done blinded.

### Transmission electron microscopy – immunogold labeling

Left ventricle heart fragments obtained from infected and healthy mice were fixed using a solution containing glutaraldehyde (1%), paraformaldehyde (4%), picric acid (0.2%), and 0.1 M sodium cacodylate for at least 3 h at 4°C. After fixation, the fragments were processed as previously described for inclusion in LR-White resin (24). Next, ultra-thin sections were obtained and transferred to collodion-coated nickel gratings. Grids were blocked with 50 mM glycine, 10% BSA (Aurion, Wageningen, Netherlands) and 0.1% Tween 20 (Sigma-Aldrich, Hamburg, Germany) for 30 minutes each. After blocking, the grids were incubated overnight at 4°C with primary anti-connexin antibody 43 (1:10; Santa Cruz Biotechnology), and then for 1 h with secondary goat anti-rabbit antibody conjugated to colloidal gold (10 nm) (1:100; Sigma-Aldrich; G7-402). For negative controls, grids were incubated in 0.1 M phosphate-buffered saline (PBS) instead of the primary antibody. All grids were finally stained with uranyl acetate and lead citrate, and subsequently examined under a JEOL TEM-1230 transmission electron microscope operating at 80Kv. The micrography analyses were done blinded.

### H9c2 cell cultures

H9c2 cells, initially isolated from an embryonic BD1X rat heart, were used to analyze Cx43 expression *in vitro* (25). H9c2 cells were cultured in Dulbecco’s modified Eagle’s medium (DMEM; Life Technologies, GIBCO-BRL, Gaithersburg, MD) supplemented with 10% fetal bovine serum (FBS; GIBCO) and 50 µg/mL of gentamicin (Life Technologies), maintained at 37°C under a humidified atmosphere of 5% CO_2_, with the culture medium changed every two days until reaching 80%-90% confluence.

H9c2 cells were plated at a density of 5x10^5^ cells per well on 24-well plates containing glass coverslips. Following cell adherence to the coverslips, cultures were stimulated with a combination of pro-inflammatory cytokines (IL-1β, TNF, and IFN-γ; 10 ng/ml of each cytokine; Cell Signaling Technology) (26, 27) in DMEM supplemented with 10% FBS for 48 h. Glass coverslips containing H9c2 cells were then washed with PBS and fixed in 4% paraformaldehyde (Thermo Scientific, Wilmington, DE) for 15 minutes at room temperature. The cells were washed again with PBS and nonspecific sites were blocked with PBS plus 5% BSA for 30 minutes and then incubated overnight at 4°C with the primary antibody, anti-total Cx43 (1:100; Santa Cruz Biotechnology). Next, the cells were washed with PBS-Tween and PBS 1x and incubated with the secondary anti-rabbit IgG antibody (AlexaFluor 488; 1:400) and Wheat Germ Agglutinin (WGA – AlexaFluor 594; 1: 1000) diluted in PBS plus 1% BSA. Slides were mounted using Fluoroshield mounting medium with DAPI (Sigma-Aldrich). Images were obtained using a TCS SP8 spectral confocal microscope (Leica), with fluorescence intensity evaluated using ImagePro software version 7.0 (Media Cybernetics).

### Dye transfer analysis

The functional assessment of gap junctions was carried out through *in vitro* dye transfer analysis. Firstly, H9c2 cells cultivated on 24-well plates at a density of 5x10^5^ cells/well were stimulated with pro-inflammatory cytokines for 48h. Incisions through monolayers were performed with the tip of a scalpel in the presence of Lucifer Yellow (LY) dye (Sigma-Aldrich) for 5 minutes. Next, cells were washed three times with PBS containing Ca^2+^ and Mg^2+^ to remove excess dye, and then fixed in 10% formaldehyde for 15 minutes at room temperature (28). Images were obtained using a ZOE Fluorescent Cell Imager (BioRad).

### Cardiomyocyte action potential and immunofluorescence analysis

Induced pluripotency stem cell (iPSC)-derived cardiomyocytes, originally collected from healthy donor, were obtained from the Laboratory of Cellular and Molecular Cardiology, Federal University of Rio de Janeiro (UFRJ) (29). iPSCs were cultured in RPMI medium (LGC Biotecnologia) supplemented with B-27 (GIBCO) without insulin. Activation of the Wnt pathway was performed by treatment with CHIR99021(9 μM), a glycogen synthase kinase 3 (GSK3) inhibitor (R&D Systems), on day 0 (D0). Subsequently, the Wnt pathway was inhibited by treatment with the antagonist XAV939 (R&D Systems) at concentrations of 10 and 5 μM, respectively on days 3 and 4 (D3 and D4). After this step, from D7 onwards, the medium initially used was replaced by medium containing insulin and RPMI/B-27 Plus, which was regularly changed every two days until complete cardiomyocyte maturation was achieved on D30. This protocol was adapted from Lian et al. (2013). The cell line Pac25 used in this work was obtained from a healthy donor was previously approved by a national ethical review board in Brazil (CONEP: # 409960/2013-6; CEP: 63167722.0.0000.5272).

Next, iPS-derived cardiomyocytes were characterized by flow cytometry. Cells were fixed and permeabilized with BD Cytofix/Cytoperm™ (BD Biosciences) and stained with pluripotency markers OCT3/4 (1:100; BD Biosciences), SOX2 (1:100; BD Biosciences), and cardiac marker Troponin T (1:100; Thermo-Fisher). (**Supplementary Figure S2**) (29).

Human iPSC-derived cardiomyocytes (3x10^5^ cells) were plated in 35 mm culture dishes in RPMI/B-27 Plus in the absence or presence of recombinant IL-1β, TNF, and IFN-γ (10 ng/ml of each cytokine) to induce an *in vitro* inflammatory microenvironment. After 24 or 48 h, cultures were used for electrophysiology analysis. Cells were continuously perfused with a Tyrode solution containing (in mM) 140 NaCl, 5.4 KCl, 1.8 CaCl2, 1.0 MgCl2, 11 D-glucose and 10 HEPES, at pH 7.4 under a constant temperature of 37°C.

Action potential was recorded using a glass microelectrode with a resistance of 40-100 MΩ (1.5 x 0.86 mm – P-97 Flaming/Brown Micropipette Puller – Sutter Instrument), filled with KCl solution (3 M) and connected to a signal amplifier (MultiClamp 700B, Molecular Devices, USA). The amplified signal was digitized (DIGIDATA 1440 A/D interface, Axon Instrument, Inc.) and the obtained data was analyzed using LabChart 7.3 software (ADInstruments, Australia). Action potential duration (APD) was analyzed at 10, 50, and 90% of repolarization, considering at least five action potentials from each group.

In addition, human iPSC-derived cardiomyocytes were also used for immunofluorescence analysis. iPSC-derived cardiomyocytes were washed with PBS following the same protocol described above for H9c2 cells. By contrast, these cells were incubated overnight at 4°C with the primary antibody anti-total Cx43 (1:100; Santa Cruz Biotechnology) diluted in PBS containing 1% BSA. Next, the cells were washed with PBS-Tween and PBS 1x and incubated with the secondary anti-rabbit antibody (AlexaFluor 568; 1:800) and phalloidin (AlexaFluor 488; 1:400) for 1 hour at room temperature. Slides were mounted using Fluoroshield mounting medium with DAPI (Sigma-Aldrich).

### Statistical analysis

Data are expressed as means ± standard error of the mean for the number of animals in each group. The normal distribution of data was confirmed by the Shapiro-Wilk test Student’s *t* test was used to compare quantitative variables between groups at a given timepoint. For comparisons between three groups, data were analyzed using one-way ANOVA, followed by the Newman-Keuls multiple comparison test. Significant differences were considered for p values below 0.05. All analyses were performed using Graph Pad Prism version 8.0 (Graph Pad Software, San Diego, CA).

## Results

### Chronic infection with *T. cruzi* reduces treadmill performance, promotes cardiac arrhythmias, inflammation and fibrosis

**Figure 1:**
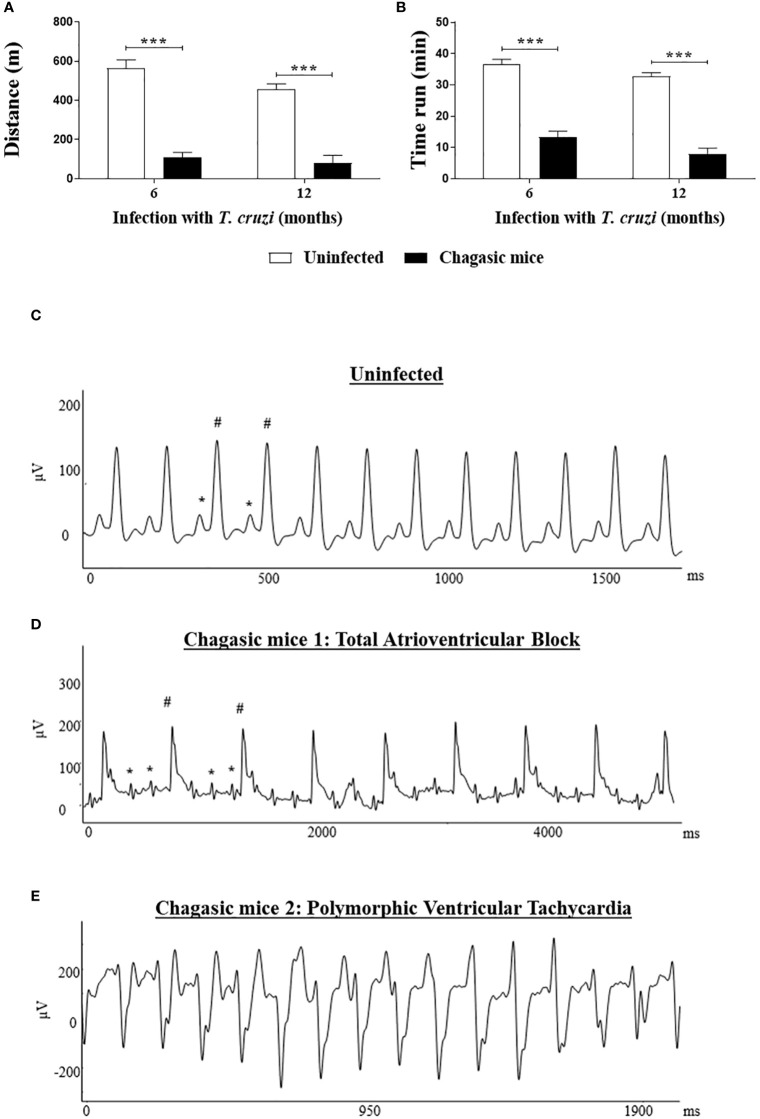
Functional analysis: Treadmill testing and EKG analysis of chagasic and uninfected mice. **(A, B)** Distance run and time of exercise on a motorized treadmill involving mice after 6 and 12 months of infection, respectively; Representative EKG images: **(C)** EKG of an uninfected mouse with regular sinus rhythm; **(D)** EKG of a chagasic mouse with total atrioventricular block; **(E)** EKG of chagasic mouse with polymorphic ventricular tachycardia. * = P wave; # = QRS complex. Treadmill test: Values represent means ± S.E.M. of 10 and 14 mice (6 months of infection) and 7 and 8 mice (12 months of infection) from uninfected and chagasic groups, respectively. ***P< 0.001 compared to uninfected group.

The cardiac function of mice with CCC was evaluated at 6 and 12 months after infection. The treadmill performance of *T. cruzi*-infected mice was significantly impaired (*P* < 0.001) compared to uninfected control littermates, which run greater distances (**Figure 1A**) for longer times of exercise (**Figure 1B**) at both times after infection. Additionally, EKG analysis revealed that *T. cruzi*-infected mice present severe cardiac conduction disturbances (**Figures 1C–E**). The alterations identified correspond to different types of arrhythmias, including polymorphic ventricular tachycardia, junctional rhythm, atrioventricular dissociation, and atrioventricular block. By contrast, uninfected control animals presented normal sinus rhythm. No significant differences were observed in the severity of arrhythmias presented by *T. cruzi*-infected mice when comparing 6 versus 12 months after infection (**Figure 1**).

**Figure 2:**
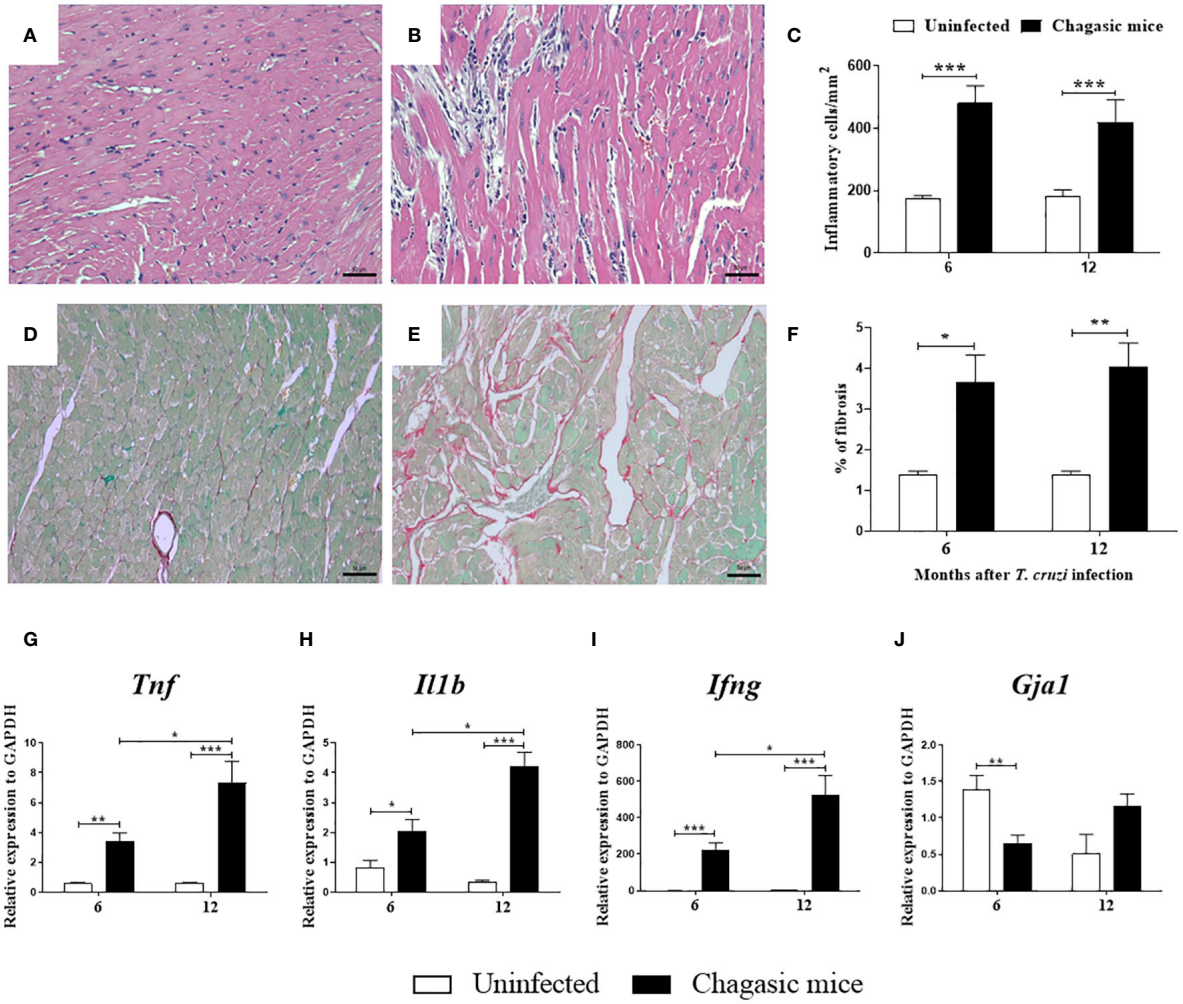
Morphological analysis and gene expression of pro-inflammatory cytokines in the hearts of uninfected and chagasic mice at 6 and 12 months after infection. **(A, B)** Representative micrographs of hematoxylin and eosin-stained heart sections of uninfected and chagasic mice at 12 months following infection. **(C)** Inflammatory cells quantified by morphometric analysis. **(D, E)** Micrographs of picrosirius red-stained heart sections of uninfected and chagasic mice. **(F)** Fibrotic area represented by percentage of collagen deposition in heart sections. Gene expression of pro-inflammatory cytokines **(G)** *Tnf*, **(H)** *Il1b*, **(I)** *Ifng*, and **(J)** *Gja1* assessed by RT-qPCR using cDNA samples prepared from mRNA extracted from experimental mouse hearts. Values represent means ± S.E.M. of 5-6 mice per group. ***P< 0.001; **P< 0.01; *P<0.05 compared to uninfected group.

Heart sections prepared from the left ventricles were stained with H&E or Sirius red for morphometric analysis of inflammation and fibrosis, respectively. An intense multifocal inflammatory infiltrate predominantly composed of mononuclear cells was found in the heart sections from *T. cruzi*-infected mice (at both 6 and 12 months). The number of inflammatory cells was significantly higher compared to uninfected controls (**Figures 2A–C**). Collagen deposition analysis indicated a greater area of fibrosis deposition in the cardiac sections of infected mice compared to controls at both time points evaluated (**Figures 2D–F**). Moreover, increased expression of genes encoding for the pro-inflammatory cytokines TNF, IL-1β, and IFN-γ was detected in heart samples obtained from the infected group compared to uninfected controls 6 or 12 months after infection (**Figures 2G–I**).

### Cardiac Cx43 gene expression, cell distribution, and phosphorylation patterns are affected by *T. cruzi* infection

To evaluate Cx43 gene expression, the left ventricles of mouse hearts were analyzed at 6 and 12 months after *T. cruzi* infection. The expression of *Gja1*, which encodes Cx43, was reduced in the hearts of chagasic mice compared to naïve mice at 6 months, but not at 12 months after infection (**Figure 2J**).

**Figure 3:**
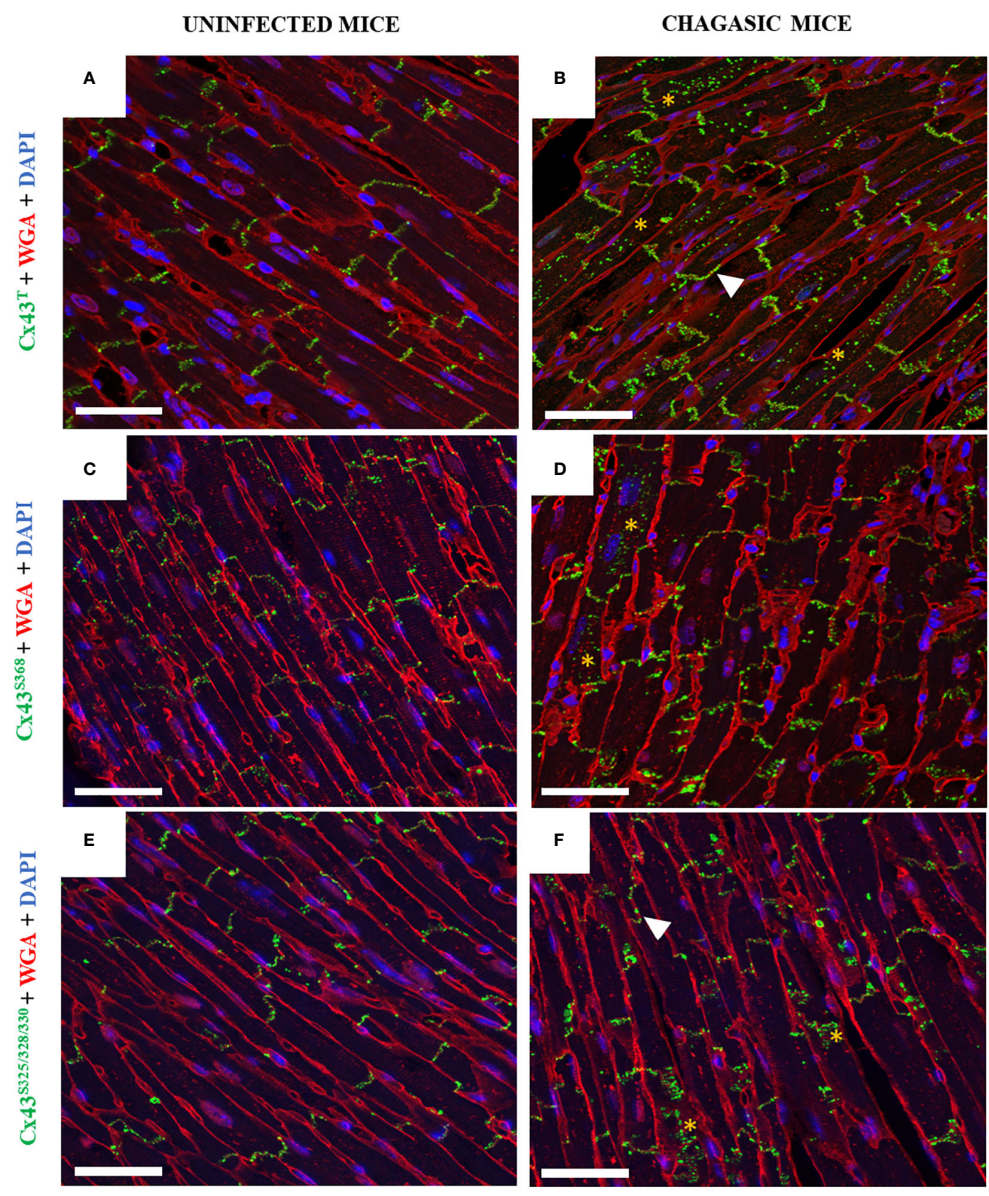
Distribution of total Cx43, Cx43^S368^, and Cx43^S325/328/330^ in hearts of chagasic mice and uninfected controls. **(A, C, E)** Heart sections from uninfected mice and **(B, D, F)** chagasic mice. Samples were stained with WGA (red), cell nuclei stained with DAPI (blue), and with anti-total Cx43, anti-Cx43^S368^, and anti-Cx43^S325/328/330^ (green) antibodies; images analyzed by confocal microscopy. Scale bars = 25 µm. White arrows indicate lateralization, while yellow asterisks indicate Cx43 in cytoplasm. WGA, Wheat Germ Agglutinin; Cx43, Connexin 43.

Next, the distribution of total Cx43 (Cx43^T^) and its phosphorylated forms (Cx43^S368^ and Cx43^S325/328/330^) were evaluated by confocal microscopy in the left ventricles. The pattern of Cx43^T^ localization was significantly altered in *T. cruzi*-infected mice compared to uninfected animals. While predominantly located in the intercalated discs in the cardiac sections of uninfected control mice (**Figure 3A**), in chagasic hearts Cx43^T^ was frequently identified outside the intercalated discs, appearing in the lateral membrane and scattered into the cytoplasm (**Figure 3B**). Similar results were observed in heart sections stained with antibodies against phosphorylated Cx43, as Cx43^S368^ and Cx43^S325/328/330^ were detected in the lateral membrane and within the cytoplasm in infected mice compared to uninfected mice, in which Cx43 was mostly restricted to intercalated discs (**Figures 3C–F**).

**Figure 4:**
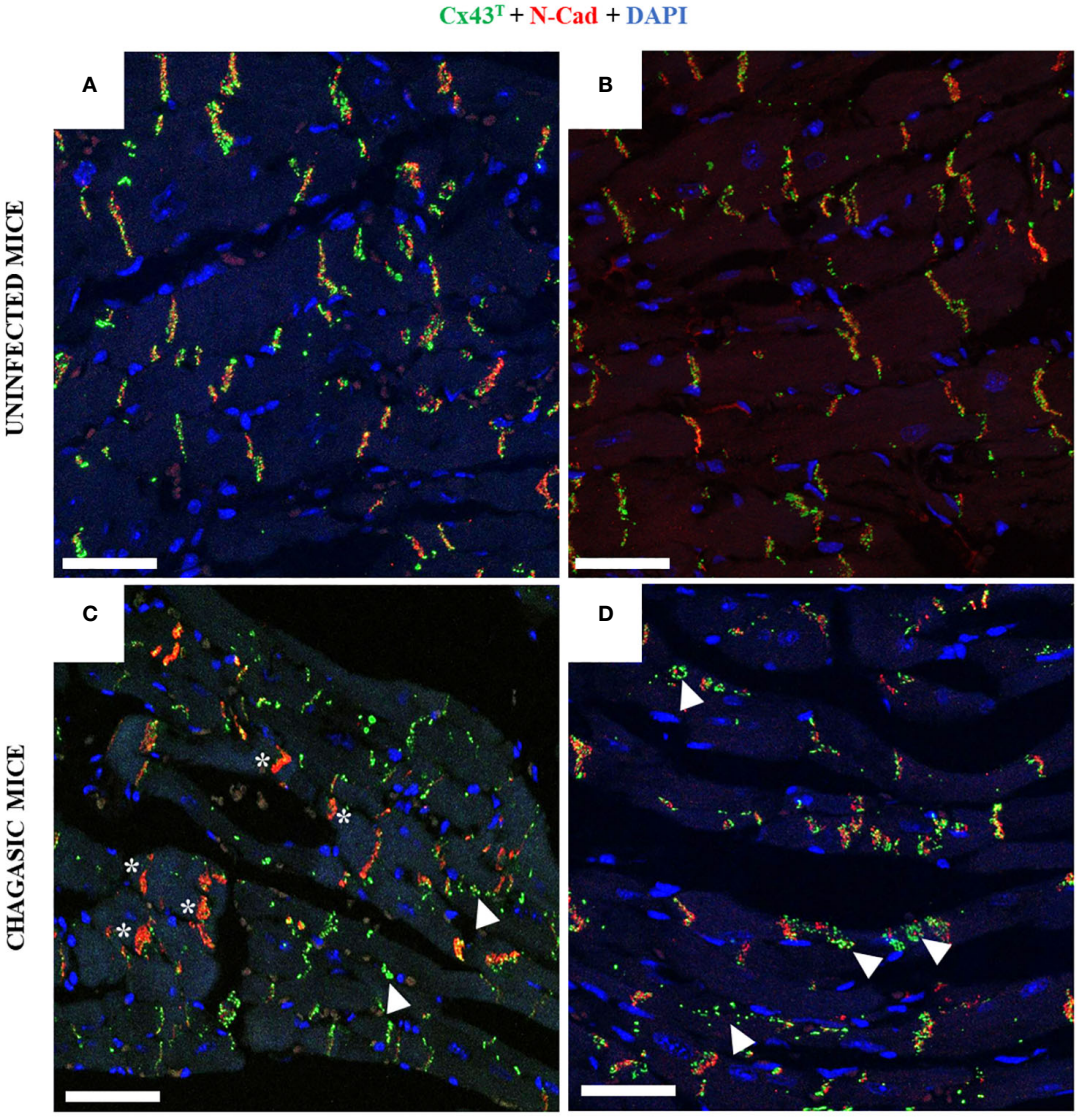
Co-staining with Cx43 and N-Cadherin in hearts of chagasic mice and uninfected controls. **(A, B)** Non-infected hearts show the presence of Cx43 (green) and N-cadherin (red) in the intercalated disk area; **(C, D)** chagasic hearts frequently presented N-cad exclusively in intercalated disc areas, with Cx43 dispersed into the cytoplasm. White arrows indicate Cx43 in cytoplasm, while white asterisks indicate intercalated discs with only N-cad expression. N-cad, N-cadherin; Cx43, Connexin 43. Scale bars = 25 µm.

Confocal microscopy analysis of heart sections co-stained with anti-Cx43^T^ and anti-N-cadherin antibodies also indicated alterations induced by chronic *T. cruzi* infection. In the hearts of uninfected animals, staining for these two proteins revealed the localization of both N-cadherin and Cx43^T^ in the intercalated discs (**Figures 4A, B**). In contrast, staining for Cx43 was weaker in the intercalated discs in chagasic mice areas, with N-cadherin almost exclusively detected in some areas. Cx43^T^ staining was often observed in areas adjacent to the intercalated disks, and also scattered into the cytoplasm as well as into lateral membranes (**Figures 4C, D**).

**Figure 5:**
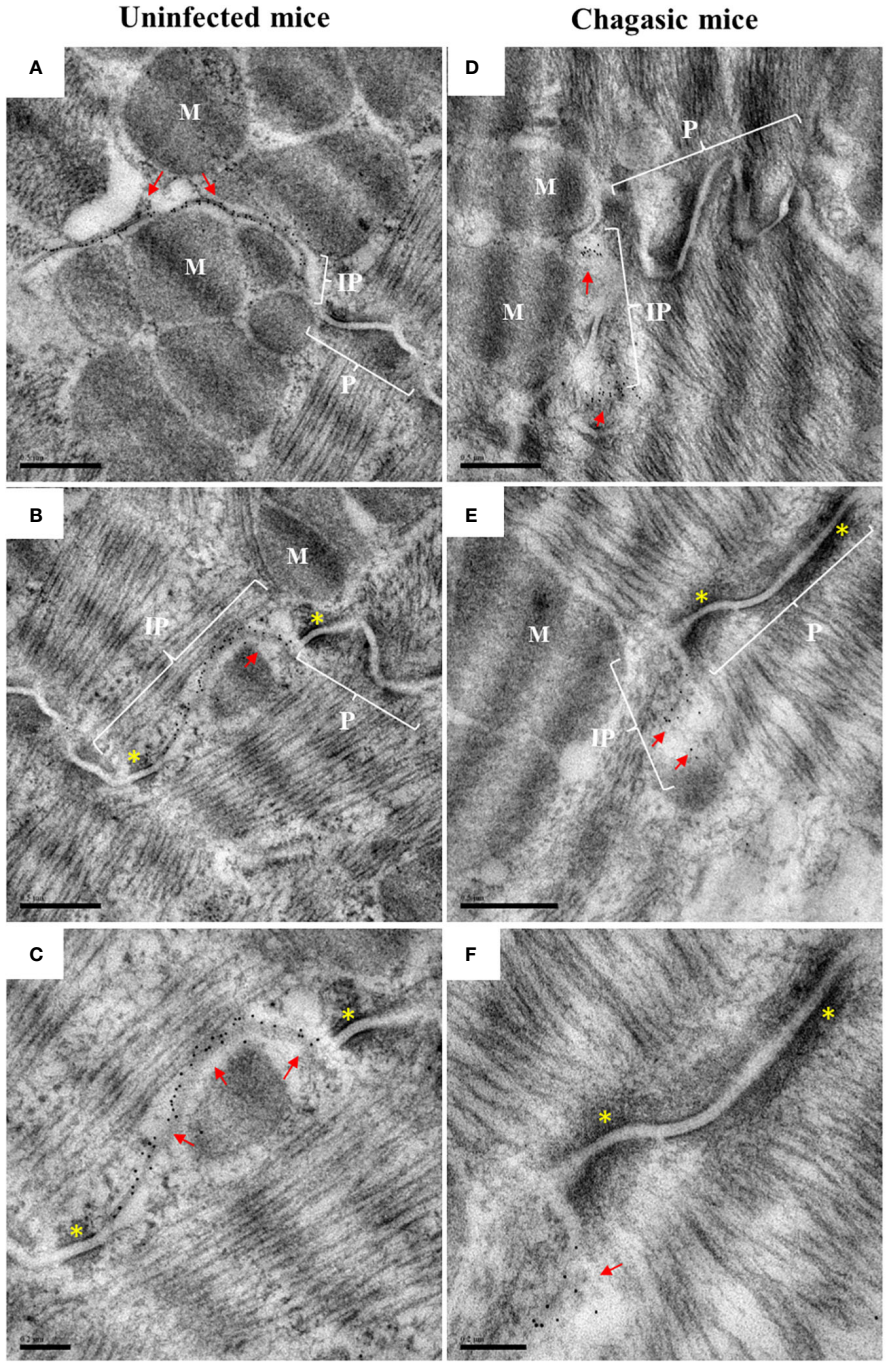
Ultrastructural analysis of total Cx43 in intercalated discs. Hearts from uninfected and chagasic control mice (n = 3 per group) were processed for analysis by transmission electron microscopy. Ultrathin sections were incubated with total anti-Cx43 antibody, with labeling revealed by the immunogold technique. **(A–C)** representative sections from uninfected control mice; **(D–F)** representative sections from chagasic mice. Red arrows indicate the presence of gap junctions in the intercalated discs; yellow asterisks indicate desmosome; P, plicate; IP, interplicate; M, myofibrils. Scale bars = 0.5 µm **(A, B, D, E)**, 0.2 µm **(C, F)**.

Next, we performed ultrastructural analysis to evaluate Cx43^T^ distribution by immunogold labeling using transmission electron microscopy in fragments of murine left ventricles. Heart samples from uninfected mice showed Cx43^T^ localized in the interplicate regions of cardiomyocyte intercalated discs, adjacent to plicate areas containing loops and desmosomes (**Figures 5A–C**). The hearts of chronic chagasic mice also showed staining for Cx43^T^ in interplicate regions (**Figures 5D–F**), as well as in invaginations adjacent to the intercalated discs (**Figure 5**).

### Cell distribution of Cx43 is altered in human chagasic hearts

**Figure 6:**
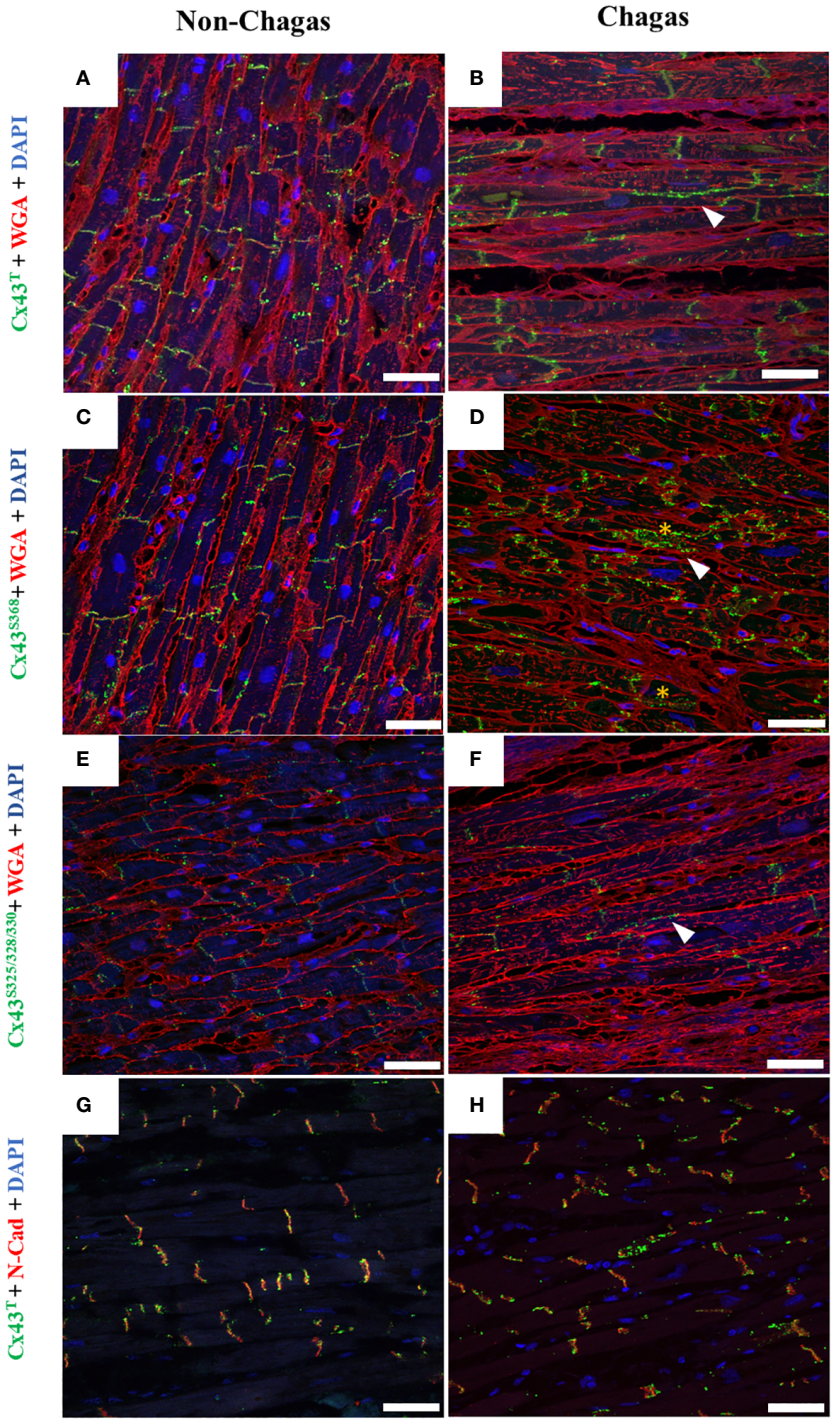
Distribution of total Cx43, Cx43^S368^, and Cx43^S325/328/330^ in human cardiac muscle tissue. **(A, C, E)** Sections of explanted hearts from non-Chagas patients and **(B, D, F)** sections of explanted hearts from Chagas patients who underwent heart transplantation were analyzed by immunofluorescence. Samples were labeled with WGA (red), cell nuclei with DAPI (blue), and total Cx43 total, Cx43^S368^ or Cx43^S325/328/330^ (green). **(G, H)** Co-labeling of Cx43 (green) and N-cadherin (red), and co-localization between Cx43 and N-cadherin (yellow). White arrows indicate the presence of Cx43 outside the intercalated disc area. Scale bars = 50 µm. WGA, Wheat Germ Agglutinin; N-cad, N-cadherin; Cx43, Connexin 43.

Samples of cardiac ventricles from patients with heart failure due to chronic Chagas disease who underwent heart transplantation were also evaluated for the distribution of Cx43^T^, Cx43^S368^, and Cx43^S325/328/330^. In a healthy heart sample, Cx43^T^ staining was predominantly localized in cardiomyocyte intercalated discs (**Figure 6A**). By contrast, Cx43^T^ localization was altered in the Chagasic hearts, as evidenced by expression in lateral membranes as well as the cytoplasm (**Figure 6B**). Using an antibody specific for Cx43^S368^, weak staining was observed in the healthy heart (**Figure 6C**) compared to intense labeling in chagasic heart sections, mainly localized in the lateral membranes and cardiomyocyte cytoplasm (**Figure 6D**). Similar results were obtained with antibodies against Cx43^S325/328/330^ (**Figures 6E, F**). Double-staining with Cx43^T^ and N-cadherin revealed the expression of both Cx43^T^ and N-cadherin in the intercalated discs of the control heart sample (**Figure 6G**). However, in chagasic hearts, less intense co-staining of Cx43^T^ and N-cadherin was seen in the intercalated disc areas, with scattered Cx43^T^ staining observed in regions adjacent to the intercalated discs (**Figure 6H**).

### A pro-inflammatory microenvironment induces alterations in Cx43 distribution, reduces dye transfer capacity, and reduces action potential duration in cardiomyocytes *in vitro*

**Figure 7:**
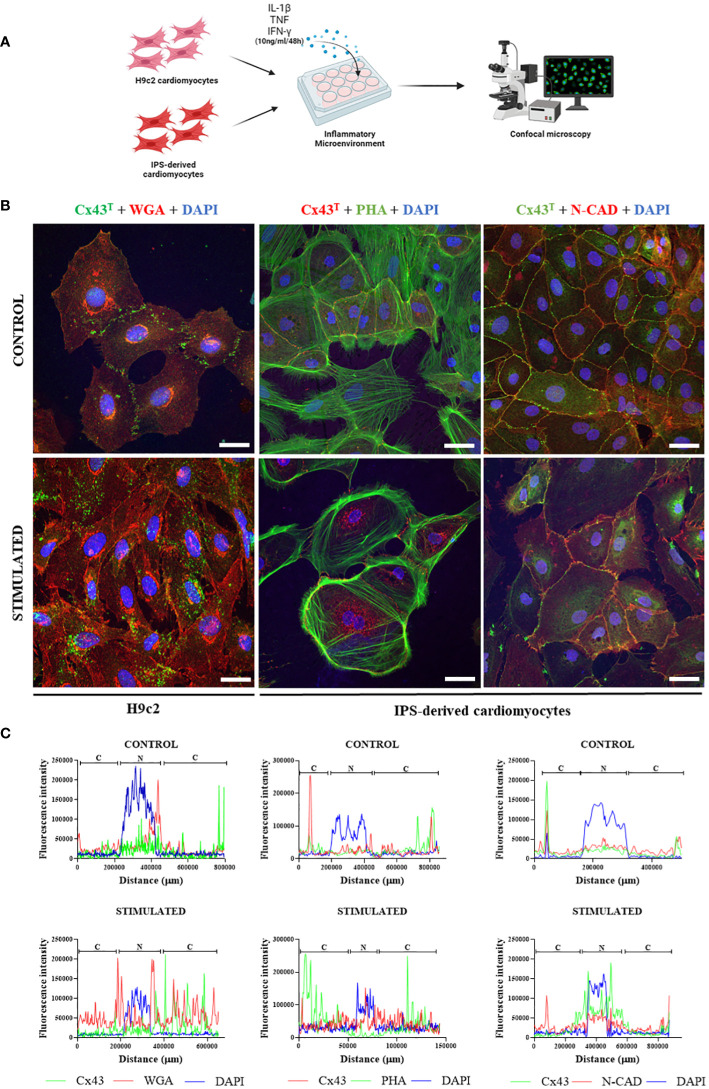
Effect of inflammatory microenvironment on Cx43 distribution *in vitro*. **(A)** Experimental *in vitro* design involving H9c2 cells and iPSC-derived cardiomyocytes, with both cell types stimulated by pro-inflammatory cytokines (IL-1β, TNF, and IFN-γ, 10 ng/ml of each cytokine) for 48 hours for immunofluorescence analysis. **(B)** Immunofluorescence of H9c2 cells and iPSC-derived cardiomyocytes; **(C)** Analysis of Cx43 distribution by fluorescence intensity in H9c2 cells and iPSC-derived cardiomyocytes after 48 hours of stimulation with pro-inflammatory cytokines IL-1β, TNF, and IFN-γ. Cells were stained with WGA (red), cell nuclei with DAPI (blue), and total Cx43 with anti-Cx43 antibody (green). Images analyzed by confocal microscopy. Scale bars = 50 µm. WGA, Wheat Germ Agglutinin; Cx43, Connexin 43; IL-1β, Interleukin 1 beta; TNF, Tumor necrosis factor; IFN-γ, Interferon gamma; C, Cytoplasm; N, Cell Nucleus. Created with BioRender.com.

IPSC-derived cardiomyocytes and H9c2 cells were stimulated *in vitro* with pro-inflammatory cytokines (IL-1β, TNF, IFN-γ) to simulate the inflammatory microenvironment found in chagasic hearts (**Figure 7A**). Confocal microscopy analysis revealed marked alterations in Cx43 distribution in both cell cultures, with staining spread throughout the cytoplasm of cardiomyocytes in contrast to non-stimulated cells, in which this protein was mainly localized in the plasma membrane region (**Figure 7B**). Immunofluorescence in H9c2 cells and iPSC-derived cardiomyocytes indicated perinuclear and dispersed Cx43 staining throughout the cytoplasm in cells stimulated with pro-inflammatory cytokines, while non-stimulated cells showed higher Cx43 fluorescence intensity in cell membranes that frequently coincided with N-cadherin staining (**Figure 7C**).

**Figure 8:**
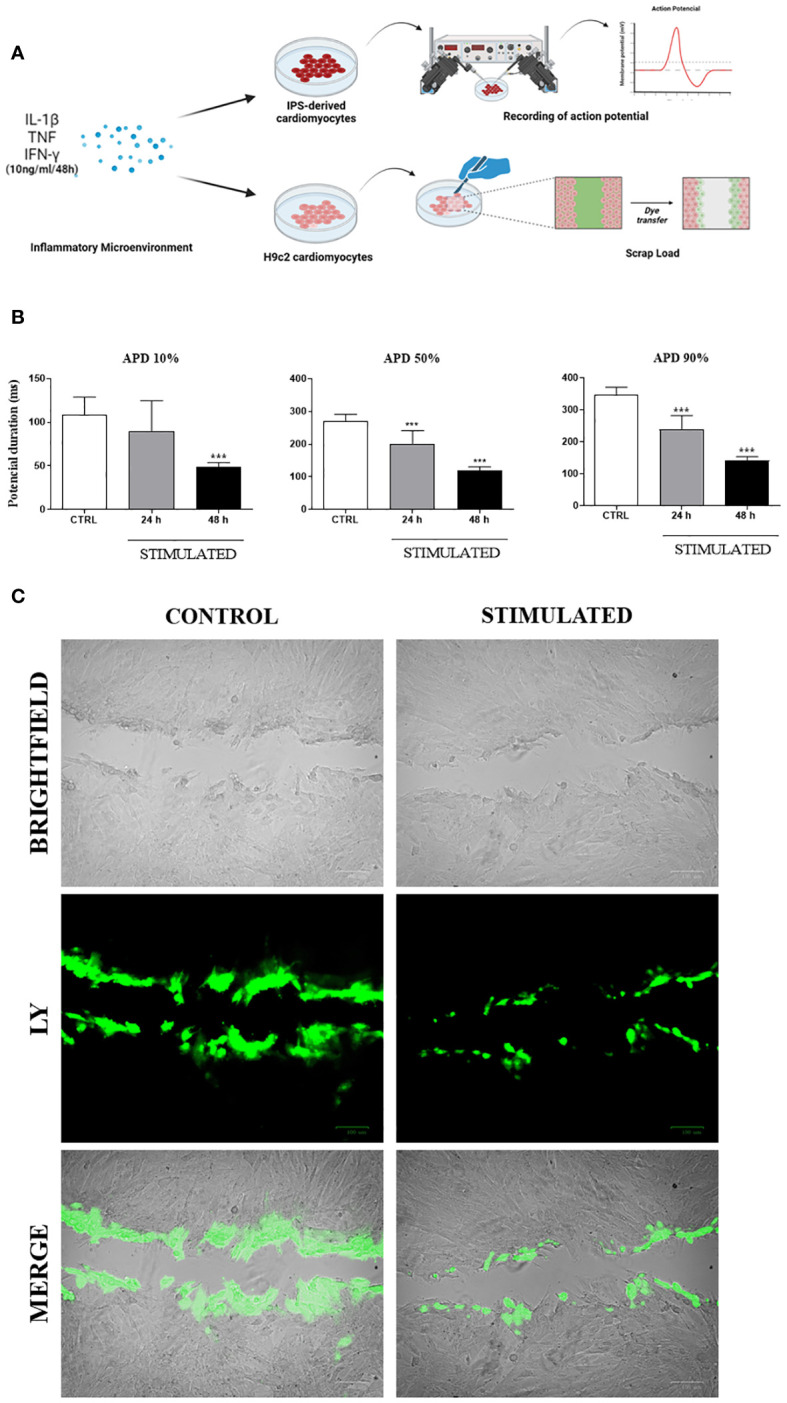
Functional *in vitro* analysis of Cx43: Action Potential Duration and Lucifer Yellow dye transfer. **(A)** Schematic drawing of functional testing performed on iPSC-derived cardiomyocytes and H9c2 cardiomyocytes, stimulated with pro-inflammatory cytokines to respectively analyze the duration of action potential and dye transfer between neighboring cells. **(B)** After stimulation, action potential duration (APD) of iPSC-derived cardiomyocytes was determined at 10%, 50% and 90% levels. **(C)** Lucifer Yellow dye transfer test (green) performed in H9c2 cells (bright field) stimulated with pro-inflammatory cytokines IL-1β, TNF, and IFN-γ for 48 hours. Predominance of dye observed in cells at the margin where scalpel incision was made, while absence of stimulation resulted in dye reaching more distant cells; IL-1β = Interleukin 1 beta; TNF = Tumor necrosis factor; IFN-γ = Interferon gamma; CTRL = Control group; ***P< 0.001 compared to unstimulated cells. Scale bars = 100 μm.

Functional analyses were performed *in vitro* to evaluate the influence of an inflammatory microenvironment on action potential duration in human iPSC-derived cardiomyocytes and dye transfer between adjacent cells in H9c2 cells (**Figure 8A**). IPSC-derived cardiomyocytes stimulated for 24 or 48 hours with a combination of pro-inflammatory cytokines showed significant reductions in APD at different repolarization points (10, 50, and 90%) compared to control cells, with a marked further reduction resulting from longer stimulation (**Figure 8B**). Finally, the extent of lucifer yellow dye transfer was assessed in H9c2 cells, revealing less diffusion of dye in stimulated cells compared to unstimulated control cells (**Figure 8C**). Dye diffusion was quantified by measuring the stained area, confirming a statistically significant difference (unstimulated cardiomyocytes: 511.8 ± 223.3 mm^2^, compared to 248.8 ± 122.9 mm^2^ in cytokine-stimulated cardiomyocytes; p = 0.015).

## Discussion

Under pathological conditions, cardiomyocyte Cx43 localization can change, contributing to improper electrical potential conduction within the heart (14, 16, 23, 30). The present study investigated alterations in conduction and correlated it with Cx43 localization in CCC. Our results indicate that CCC is associated with marked alterations in Cx43 distribution and phosphorylation patterns in an experimental mouse CCC model. Importantly, our analysis of human heart samples obtained from end-stage CCC subjects revealed similar alterations in Cx43 distribution. Moreover, *in vitro* experimentation mimicking the inflammatory microenvironment found in CCC also induced alterations in Cx43 distribution in both human and murine cardiomyocytes, as well as impaired intercellular communication through gap junctions. Taken together, these results reinforce the role played by inflammation in promoting structural and functional cardiac alterations, as well as highlighting the potential of immunomodulation as a potential strategy for the treatment of CCC (31).

Mice with CCC exhibited impaired cardiac function, as evidenced by electrical conduction disturbances, which is consistent with previous studies employing a similar experimental approach (20, 21, 32). Our EKG analysis revealed different degrees of arrhythmias in animals with CCC, which is also convergent with previous reports (20, 32, 33). Additionally, mice with CCC exhibited a reduced ability to run on a treadmill, which could be associated with a compromised cardiac function (20, 32), as well as skeletal muscle alterations, since chronic *T. cruzi* infection is also characterized by intense myositis (34).

Intense inflammation and fibrosis, hallmarks of chronic Chagas cardiomyopathy, were identified in the hearts of chronically infected mice, similarly to reports in previous studies (20, 21, 32). Both processes may contribute to the development of the conduction disturbances observed in our model. Fibrosis deposition constitutes an important factor in the genesis of arrhythmias, suppressing the propagation of cardiac action potential (35). At the same time, a persistent inflammatory process leads not only to the loss of myofibers and the replacement of contractile fibers with fibrosis, but is also responsible for neuronal destruction of the autonomic nervous system that may contribute to the genesis of conduction disturbances (36).

Phosphorylation at different serine and/or tyrosine residues in the C-terminal domain of Cx43 plays an important role in the regulation of this protein’s biological activity, causing electrical decoupling in the heart, which may lead to reentry circuits and enable the onset of ventricular arrhythmias (5, 6, 37–39). Here, we observed alterations in the localization of Cx43^T^, Cx43^S368^, Cx43^S325/328/330^ in both the experimental animal model as well as in human hearts. Altered staining patterns for Cx43 isoforms were observed in the lateral membrane and internalized in the cytoplasm of cardiac cells. Previous studies have shown that the localization of Cx43 outside the intercalated disks reduces the speed of the action potential propagation (40). Conversely, in the hearts of uninfected mice, Cx43 was predominantly localized in the intercalated discs, which is indicative of effective “cell-cell” conduction of electrical potential between neighboring cardiomyocytes (15, 41–43). The increased phosphorylation of Cx43^S368^ is known to reduce permeability and promote lateralization and internalization of communicating channels, a phenomenon observed in experimental models of cardiac ischemia (18, 44, 45). Moreover, Lampe et al. (2006) suggested that the dephosphorylation of Cx43^S325/328/330^ plays an important role in compromising permeability in gap junctions, reducing communication between cardiomyocytes, in the context of heart disease, which may also be occurring in CCC.

Inflammation is known to induce alterations in the gap junctions formed by Cx43 by impairing protein expression and localization (5, 20, 21). Elevated levels of pro-inflammatory cytokines, such as TNF, IL-1β, and IFN-γ, have been shown to promote alterations in Cx43 expression (15, 27, 46–48), as well as increase Cx43^S368^ phosphorylation by activating different signaling pathways (7, 13, 15, 18, 46, 49). These observations underscore the significance of our findings in this study, in which increased expression of genes encoding cytokines IL-1β, TNF and IFN-γ was observed in the hearts of mice with CCC. The correlation between inflammatory cytokine production and changes in Cx43 phosphorylation and localization observed in our study further highlights the intricate interplay between inflammation and cardiac electrical coupling in the context of CCC.

Alterations in intercalated disc structure influence the regulation of cardiac conduction and are associated with pathological conditions (50). To better investigate the distribution of Cx43 in intercalated discs, we performed co-staining with an antibody against N-cadherin (a protein present in intercalated discs) along with Cx43. This analysis revealed scarce staining for Cx43 in the intercalated discs of chagasic heart sections, whereas uninfected mice exhibited more uniform and correlated staining for N-cadherin and Cx43 in the intercalated discs. This alteration in distribution pattern aligns with findings reported by Himelman and colleagues in a model of Duchenne muscular dystrophy (23). Alterations in Cx43 distribution were further confirmed by transmission electron microscopy, which demonstrated the localization of Cx43 in the interplicate regions of intercalated discs, as well as frequent labeling in concentric ring structures, which is suggestive of alterations in gap junctions containing membranes during internalization. Similar ultrastructural alterations were also reported by Hesketh and collaborators (2010), who evaluated Cx43 distribution in canine models of heart failure (41).

The present study observed significantly lower expression of the gene that encodes Cx43 (*Gja1*) in mice at six months after infection, similarly to previous studies reporting the downregulation of Cx43 in the heart (45, 51). However, at 12 months after infection, higher *Gja1* expression was noted, which may indicate the presence of a compensatory mechanism in response to the cardiac damage caused during disease progression (51). Further analysis will be necessary to fully clarify the long-term regulation of Cx43 gene expression within the context of CCC.

To further elucidate the influence of inflammation on Cx43 expression and function, we simulated an inflammatory microenvironment in cultures of human iPSC-derived cardiomyocytes and H9c2 cells by incubation with a pool of pro-inflammatory cytokines (IL-1β, TNF and IFN-γ). After 48 hours of stimulation, a reduction in the duration of action potential in iPSC-derived cardiomyocytes was noted, as well as the decreased ability of H9c2 cells to transfer LY dye between neighboring cells and greater Cx43 dispersion throughout the cytoplasm in both cell types investigated. Previous studies demonstrated that *T. cruzi*-infected cardiomyocytes lose the ability to transfer LY dye to adjacent cells (9, 48, 52–54). Taken together, our results indicate that the persistence of a pro-inflammatory microenvironment due to scarce residual parasitism during the chronic phase of infection may also play a major role in Cx43 disorganization (10, 12, 55), as well as contribute to the conduction disturbances seen in CCC.

In conclusion, the pro-inflammatory milieu associated with CCC provokes a marked impairment in Cx43 distribution and cardiac function. The present findings suggest possibilities for further investigations to seek a deeper understanding of the signaling pathways that may be activated by pro-inflammatory cytokines, resulting in the dysregulation of Cx43. Additionally, we highlight the importance of exploring potential therapeutic targets that could improve Cx43 function and prevent the development of serious arrhythmias in patients with CCC.

## Data availability statement

The raw data supporting the conclusions of this article will be made available by the authors, without undue reservation.

## Ethics statement

The studies involving humans were approved by the National Ethical Review Board in Brazil (CONEP). The studies were conducted in accordance with the local legislation and institutional requirements. The participants provided their written informed consent to participate in this study. The animal study was approved by Animal Use Ethics Committee of the São Rafael Hospital and the Gonçalo Moniz Institute. The study was conducted in accordance with the local legislation and institutional requirements.

## Author contributions

BB: Conceptualization, Data curation, Formal analysis, Investigation, Methodology, Writing – original draft, Writing – review & editing. MN: Data curation, Formal analysis, Methodology, Writing – original draft. CC: Formal analysis, Methodology, Writing – original draft, Data curation. CM: Conceptualization, Data curation, Formal analysis, Writing – original draft, Writing – review & editing, Methodology. PD: Data curation, Formal analysis, Methodology, Writing – original draft. CF: Data curation, Formal analysis, Methodology, Writing – original draft. GS: Formal analysis, Methodology, Writing – original draft, Data curation. DS: Formal analysis, Methodology, Writing – original draft, Data curation. FT: Formal analysis, Methodology, Writing – original draft. JN: Formal analysis, Methodology, Writing – original draft. SM: Conceptualization, Data curation, Formal analysis, Investigation, Methodology, Writing – original draft. PL: Formal analysis, Methodology, Writing – original draft. KC: Formal analysis, Methodology, Writing – original draft, Data curation. TKB: Formal analysis, Methodology, Writing – original draft, Data curation. RR: Conceptualization, Formal analysis, Funding acquisition, Supervision, Writing – original draft. AC: Conceptualization, Formal analysis, Funding acquisition, Project administration, Supervision, Writing – original draft, Writing – review & editing. MS: Conceptualization, Data curation, Formal analysis, Funding acquisition, Project administration, Supervision, Writing – original draft, Writing – review & editing.
