# Supplementary material for: The effects of inflammation on connexin 43 in chronic Chagas disease cardiomyopathy
Source: Front Immunol. 2024 Jul 29;15:1440662. doi: 10.3389/fimmu.2024.1440662 (PMC11317259; doi:10.3389/fimmu.2024.1440662)
Supplement: Supplementary file 1 [file DataSheet_1.pdf]

Supplementary Material

The effects of inflammation on connexin 43 in chronic Chagas disease cardiomyopathy

Breno Cardim Barreto[1,2,3], Maria Vitória Gomes das Neves[1,2], Carine Machado Azevedo Cardoso[1], Cássio Santana Meira[1,3], Pâmela Santana Daltro[2], Cláudio Pereira Figueira[1], Girlaine Café Santos[1,2], Daniela Nascimento Silva[3], Fábio Távora[4], João David de Souza Neto[4], Simone Garcia Macambira[1,2], Paul D. Lampe[5], Keyla Cristiny da Silva Coutinho[6], Tais Hanae Kasai Brunswick[6], Ricardo Ribeiro dos Santos[1,3,ǂ], Antônio Carlos Campos de Carvalho[6], Milena Botelho Pereira Soares[1,3*]

[1]Gonçalo Moniz Institute, Oswaldo Cruz Foundation (IGM-FIOCRUZ/BA), Salvador, Brazil;

[2]Federal University of Bahia (UFBA), Salvador, Bahia, Brazil;

[3]SENAI Institute of Innovation in Health Advanced Systems (CIMATEC ISI SAS), University Center SENAI/CIMATEC, Salvador, Bahia, Brazil;

[4]Messejana Hospital, Fortaleza, Ceará, Brazil;

[5]Fred Hutchinson Cancer Center, Seattle, WA, USA;

[6]Biophysics Institute Carlos Chagas Filho, Federal University of Rio de Janeiro, Rio de Janeiro, Brazil;

[ǂ]In memoriam

[*]Corresponding author: Milena Botelho Pereira Soares. Instituto Gonçalo Moniz, Fundação Oswaldo Cruz. Rua Waldemar Falcão, 121, Candeal, Salvador, Bahia, Brazil. Zip code: 40296-710. Phone: (+55) 71 31762260; fax: (+55) 71 31762272. Email address: milena.soares@fiocruz.br

Supplementary Figures

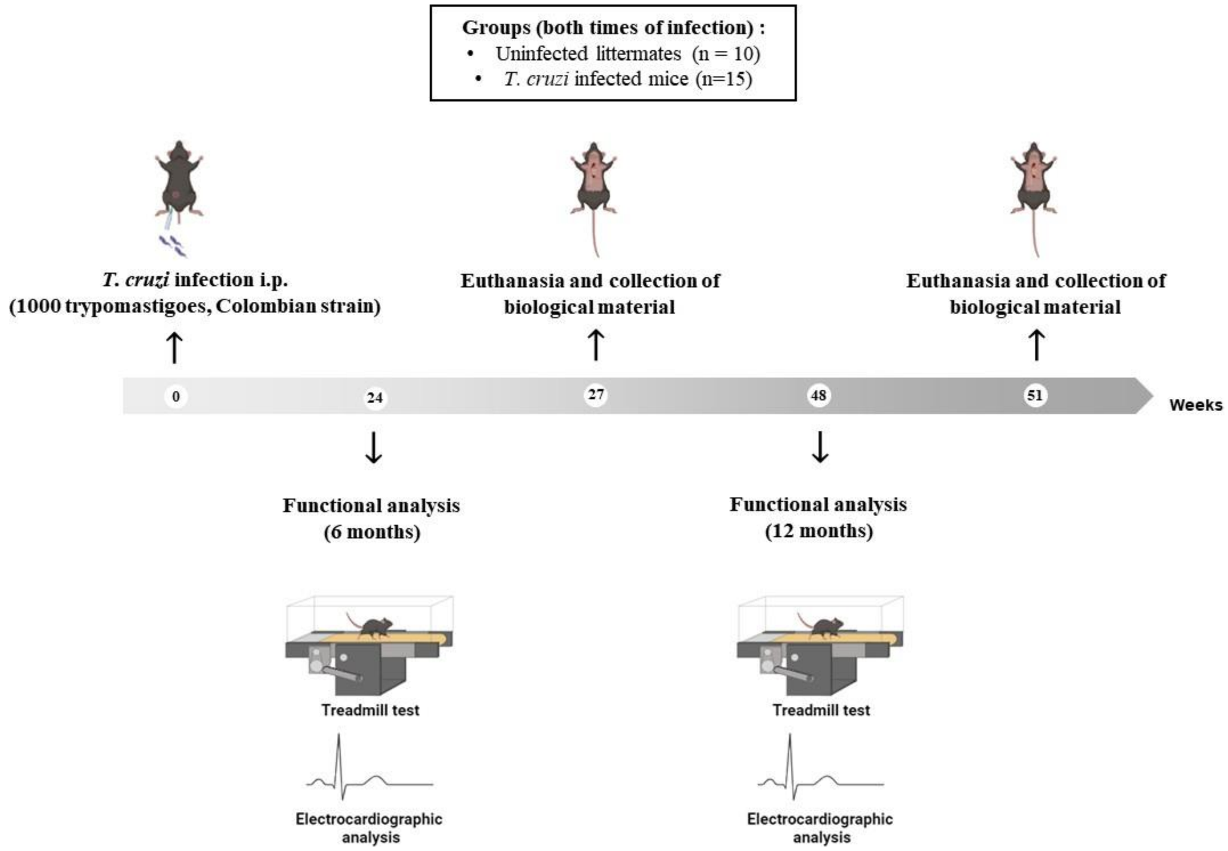

Figure S1. Experimental design: C57BL/6 mice were infected via i.p with 1000 trypomastigotes of *T. cruzi* (Colombian strain), and after 24 and 48 weeks the animals were subjected to functional analyzes (ECG and exercise test) and euthanized for collection of biological materials in weeks 27 and 51, respectively.

Figure S2

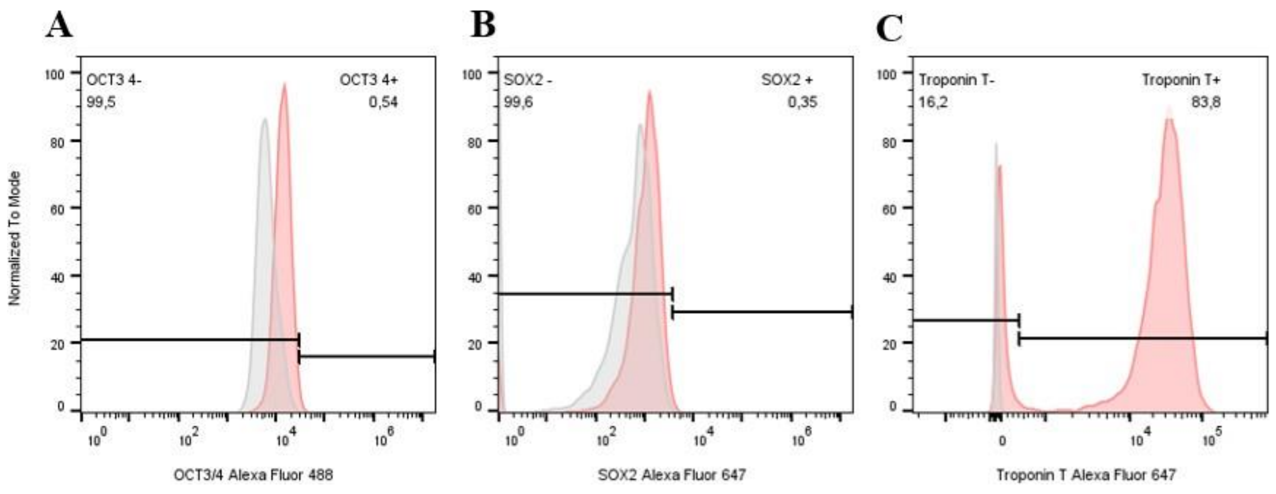

Figure S2. Flow cytometry characterization of iPSC-derived cardiomyocytes. (**A-B**) Cells were evaluated for the expression of pluripotent markers OCT3/4 and SOX2 respectively; (**C**) and cardiac protein (Troponin T). Overlay histograms representative of non-stained cells - negative control (gray) - and stained cells (red). The percentage of positive and negative cells for each marker are indicated inside the graphs.

Supplementary Table

Table S1. Characteristics of patients from which cardiac samples were obtained.

Patient	Group	Sex	Age	Description
AP345	Non-Chagas	Man	70 years	Patient without significant cardiomyopathy who died of stomach cancer
AP1065	Chagas	Man	45 years	Patient with dilated cardiomyopathy associated with chronic inflammatory cardiomyopathy
AP829	Chagas	Man	63 years	Patient with dilated cardiomyopathy associated with chronic inflammatory cardiomyopathy
AP1617	Chagas	Man	63 years	Patient with dilated cardiomyopathy, thrombus in the left ventricular wall, mild interstitial fibrosis
